# *Gardnerella vaginalis* Subgroups Defined by *cpn*60 Sequencing and Sialidase Activity in Isolates from Canada, Belgium and Kenya

**DOI:** 10.1371/journal.pone.0146510

**Published:** 2016-01-11

**Authors:** John J. Schellenberg, Teenus Paramel Jayaprakash, Niradha Withana Gamage, Mo H. Patterson, Mario Vaneechoutte, Janet E. Hill

**Affiliations:** 1 Department of Veterinary Microbiology, University of Saskatchewan, Saskatoon, Canada; 2 Laboratory for Bacteriology Research, Ghent University, Ghent, Belgium; Rush University, UNITED STATES

## Abstract

Increased abundance of *Gardnerella vaginalis* and sialidase activity in vaginal fluid is associated with bacterial vaginosis (BV), a common but poorly understood clinical entity associated with poor reproductive health outcomes. Since most women are colonized with *G*. *vaginalis*, its status as a normal member of the vaginal microbiota or pathogen causing BV remains controversial, and numerous classification schemes have been described. Since 2005, sequencing of the chaperonin-60 universal target (*cpn*60 UT) has distinguished four subgroups in isolate collections, clone libraries and deep sequencing datasets. To clarify potential clinical and diagnostic significance of *cpn*60 subgroups, we undertook phenotypic and molecular characterization of 112 *G*. *vaginalis* isolates from three continents. A total of 36 subgroup A, 33 B, 35 C and 8 D isolates were identified through phylogenetic analysis of *cpn*60 sequences as corresponding to four “clades” identified in a recently published study, based on sequencing 473 genes across 17 isolates. *cpn*60 subgroups were compared with other previously described molecular methods for classification of *Gardnerella* subgroups, including amplified ribosomal DNA restriction analysis (ARDRA) and real-time PCR assays designed to quantify subgroups in vaginal samples. Although two ARDRA patterns were observed in isolates, each was observed in three *cpn*60 subgroups (A/B/D and B/C/D). Real-time PCR assays corroborated *cpn*60 subgroups overall, but 13 isolates from subgroups A, B and D were negative in all assays. A putative sialidase gene was detected in all subgroup B, C and D isolates, but only in a single subgroup A isolate. In contrast, sialidase activity was observed in all subgroup B isolates, 3 (9%) subgroup C isolates and no subgroup A or D isolates. These observations suggest distinct roles for *G*. *vaginalis* subgroups in BV pathogenesis. We conclude that *cpn*60 UT sequencing is a robust approach for defining *G*. *vaginalis* subgroups within the vaginal microbiome.

## Introduction

*Gardnerella vaginalis* is recognized as a ubiquitous element of the complex of vaginal organisms in healthy women, but is more abundant in women diagnosed with bacterial vaginosis (BV), a poorly defined and recalcitrant clinical entity affecting reproductive health [1, 2]. The phenotypic heterogeneity of *G*. *vaginalis* is well known, and several biotyping and genotyping methods have been developed [3, 4]. For example, amplified ribosomal DNA restriction analysis (ARDRA) was introduced in 1997 [4] and has been applied to classify *G*. *vaginalis* into subgroups in several studies [5, 6]. More recent sequence-based analyses have demonstrated that this taxon consists of four distinct molecular subgroups, likely to be different species, based on 473 genes common to 17 *G*. *vaginalis* isolates (clades 1–4) [7], or by sequencing a single 552 bp region of the chaperonin-60 (*cpn*60) gene (subgroups A-D) [8]. Real-time PCR assays based on clade-specific genes and designed to quantify all four clades simultaneously in vaginal samples have shown associations between specific clades of *G*. *vaginalis* and BV in a study of 60 women in the United States with chronic vaginal symptoms [9]. It has not yet been determined whether these classifications are consistent with each other or whether clinically significant phenotypic characteristics, such as sialidase activity [10] and biofilm formation [11, 12] are differentially distributed among subgroups.

Sialidase activity is an important virulence factor associated with mucin degradation in BV and aerobic vaginitis [13], contributing to adverse pregnancy outcomes [10]. Although this activity is commonly detected in *G*. *vaginalis*, the trait is not common to all isolates, including the type strain (ATCC 14018), and expression levels are highly variable among sialidase positive isolates [14]. Although some recent studies have investigated the presence of a putative sialidase gene in relation to sialidase activity and ARDRA subgroups [5, 6], the relationship between sialidase activity and more recent genotyping techniques has not been resolved. Therefore, the objectives of the current study were: 1) to reconcile *cpn*60 UT-based molecular subgroups A-D with previously published clades 1–4, 2) to define *cpn*60 UT subgroups of 112 *Gardnerella* isolates and compare to classification based on ARDRA and clade-specific real-time PCR assays and 3) to determine sialidase gene presence and activity in all 112 isolates, in order to clarify distribution of this virulence factor across *G*. *vaginalis* subgroups.

## Methods

### Bacterial cultures and DNA extraction

The type strain of *G*. *vaginalis* (ATCC 14018) was obtained from the American Type Culture Collection. All other isolates were obtained from previous studies of women from Kenya, Canada and Belgium, as previously described [5, 8, 15]. Freezer stocks in 4% (w/v) skim milk or NYC medium (ATCC broth #1685) with 10% glycerol (v/v) were revived on Columbia 5% sheep’s blood agar (CBA; BD Biosciences, Mississauga, ON) and incubated anaerobically at 37°C using GasPak EZ sachets (BD Biosciences, Mississauga, ON) in sealed jars for 48–72h. After two passages, isolates were harvested with a sterile swab into 2 ml sterile saline (0.85% NaCl, pH 7.4) until turbidity was equivalent to McFarland standard 4. Turbidity was also assessed quantitatively by measuring optical density of 100 μl harvested cultures in duplicate wells of optically clear 96-well plates at 595 nm in a Vmax microplate reader (Molecular Devices, Inc., Sunnyvale, CA). DNA was isolated from 100 μl of harvested culture by resuspending in a 5% solution of Chelex (Bio-Rad Inc., Mississauga, ON), followed by incubation at 60°C for 30 min, 100°C for 8 min, and supernatant used for all described PCR assays.

### Determination of *cpn*60 subgroup and phylogenetic analysis

*cpn*60 subgroup (A, B, C or D) for all isolates included in this study was determined by Sanger sequencing as previously described [8]. Phylogenetic comparison was carried out including *cpn*60 universal target sequences extracted from the *cpn*60 database, cpnDB (www.cpndb.ca) [16], as well as all available whole genome sequences in the Integrated Microbial Genomes Expert Review (IMG/ER) database [17]. Full-length 16S rRNA gene sequences were available for 18 of the 36 whole genome sequences in GenBank [18]. Neighbour-joining phylogenetic trees for *cpn*60 and 16S rRNA gene sequences were created based on ClustalW aligned sequences in MEGA v6 software, with bootstrap calculations based on 100 replicates. For whole genome sequences, Pairwise Average Nucleotide Identity values using the MUMmer algorithm and Tetranucleotide correlations were calculated within and between subgroups using JSpecies [19]. Density plots were created using regular ggplot2 functions in R [20].

### Amplified ribosomal DNA restriction analysis (ARDRA)

Amplification of the 16S rRNA gene, digestion of amplicon using *Taq*I restriction enzyme and electrophoresis were carried out as described previously [8], based on techniques previously used to define genotypes of *G*. *vaginalis* [4, 5]. Primer sequences are provided in S1 Table.

### Real-time PCR to detect *G*. *vaginalis* subgroups

All isolates were assessed using SYBR Green and hydrolysis probe assays that were previously designed to detect *G*. *vaginalis* subgroups in vaginal samples based on available whole genome sequences defining clades 1 to 4 [9]. In this study, these primer/probe sets were used to assess isolates by real-time PCR. Reactions were carried out in a CFX96 thermal cycler (Bio-Rad Inc., Mississauga, ON), using previously described reaction mixtures and PCR conditions [9]. Isolates were defined as positive when the average fluorescence value (relative fluorescence units or RFU) of the last 10 cycles of the amplification reaction, minus the standard deviation, was greater than 800 RFU. Primer and probe sequences are provided in S1 Table.

### Detection of sialidase gene presence

Presence of the putative sialidase A gene was assessed using previously published primer sets (S1 Table). For conventional PCR, primers Sia1-F and -R were applied as previously described [6]. For real-time PCR using SYBR Green, primers GVSI-F and -R were applied as previously described [5]. Positive isolates were defined as described above. Differences in sialidase gene presence were evaluated by Fisher's exact test in R [20].

### Quantification of sialidase activity

Initially, a qualitative filter paper spot test was applied to detect sialidase enzyme activity of *G*. *vaginalis* isolates, as previously described [8]. Subsequently, a more sensitive assay using quantitative fluorometry was applied [14]. The fluorogenic substrate for both assays was 2'-(4-methylumbelliferyl)-α-D-N-acetylneuraminic acid sodium salt hydrate (Sigma-Aldrich Canada, Oakville, ON) dissolved in water (0.015% w/v) and aliquots stored at -20°C. Prior to the assay, aliquots of substrate were thawed and 9 parts diluted with 1 part 1 M sodium acetate (pH 5.8) and 10 μl of the reaction mixture was applied to filter paper circles. Cells harvested from blood plates as described above (10 μl of McFarland standard 4 in saline) were added to each circle and incubated at 37°C for 30 min in the dark. Sialidase activity was determined by visualizing filter paper under UV light. For quantitative assays, 100 μl of substrate was combined with 50 μl culture harvested from plates as described above into duplicate wells of a black polystyrene flat bottom microplate (Whatman Inc., Clifton, NJ), prior to measuring RFU over time in a FLx800 fluorometer (BioTek Inc., Winooski, VT). Readings were taken every 2 min. after 6 seconds of shaking at 30°C over a 30 min. period. The rate of substrate conversion in positive isolates was expressed as the increase in RFU over time, adjusted for cell concentration as measured using McFarland standards and OD_595_ as described above. Differences in sialidase activity across subgroups were evaluated by non-parametric Kruskal-Wallis and Mann Whitney U tests in SPSS (IBM Inc., Chicago, Illinois).

## Results

### Reconciling *cpn*60 UT subgroups with other published subgroups

**Fig 1:**
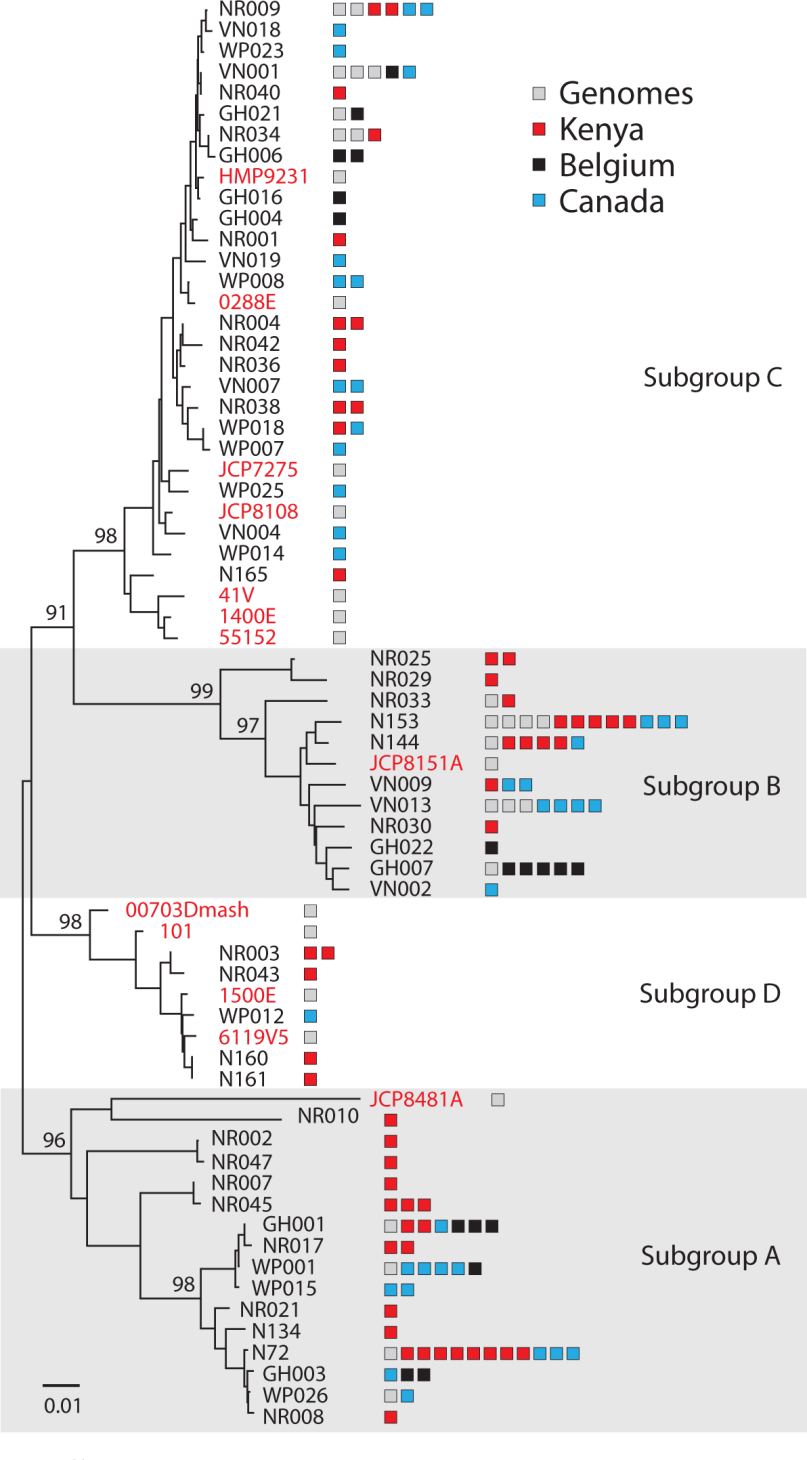
Phylogenetic relationships of *G*. *vaginalis* study isolates. Neighbour-joining phylogenetic tree of nucleotide sequences (alignment length = 552 bp) of 68 *cpn*60 sequences representing 112 isolates and 36 other isolates for which *cpn*60 sequence was available from published genomes, using ClustalW and 100 bootstrap replicates in MEGA v6. *cpn*60 sequences were collapsed to 68 unique branches using blastclust (100% identical over 100% length). Representative sequences from study isolates are in black text, while sequences extracted from published genomes are shown in red. For each branch, genomes and isolates with identical *cpn*60 sequences are shown as adjacent boxes with colour based on origin (grey = published genomes, blue = Canada, red = Belgium, black = Kenya).

In order to determine whether our previously published subgroups (A-D) correspond to previously published clades (1–4), we compared *cpn*60 UT sequences for 112 *G*. *vaginalis* isolates from three continents with *cpn*60 UT sequences extracted from previously published whole genome sequences (Fig 1). All new isolates and previously published genomes fall into four *cpn*60 subgroups [8], including 17 isolates defined as belonging to clades 1–4 [7]. These results indicate that similar phylogenetic resolution can be achieved using a partial single gene sequence (552 bp), compared to using 473 full-length gene sequences common to all 17 genomes. *cpn*60 subgroup A corresponds to clade 4, subgroup B to clade 2, subgroup C to clade 1, and subgroup D to clade 3.

**Fig 2:**
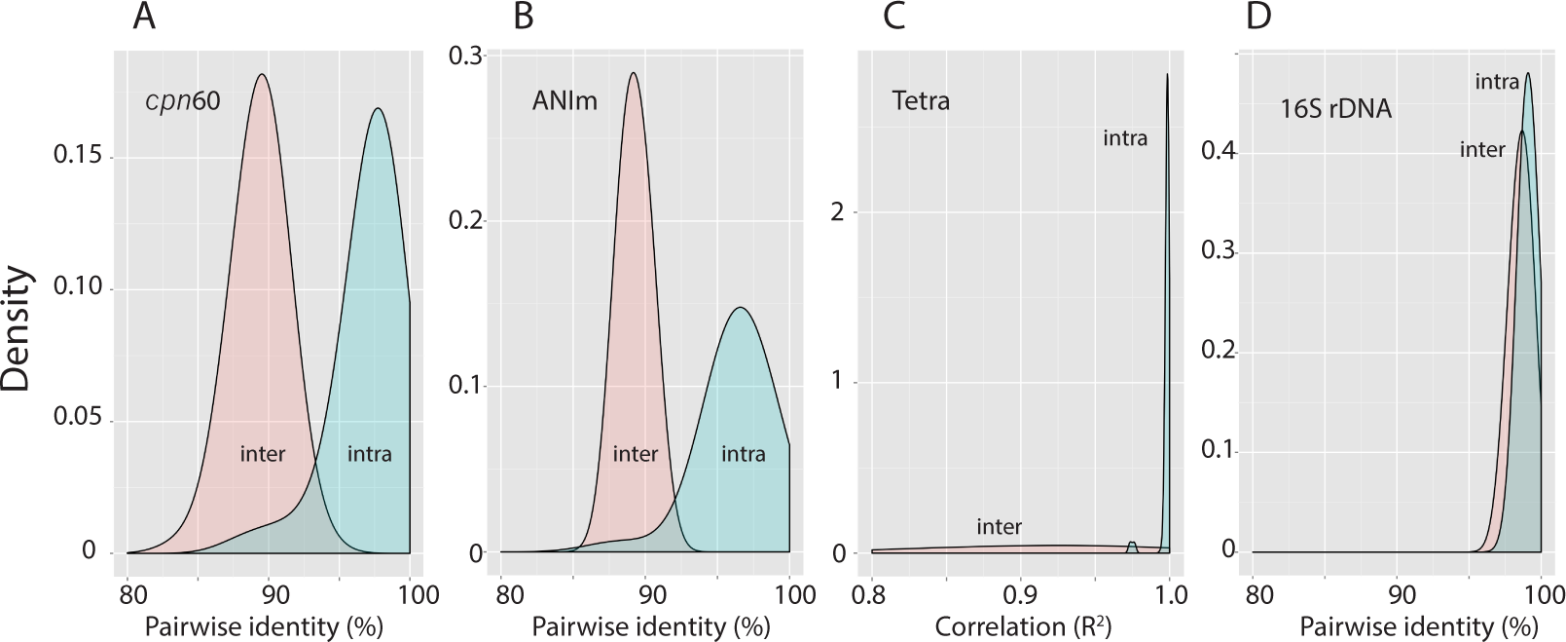
Intra- and inter-subgroup relationships based on *cpn*60 and whole genome sequences. Density plots of pairwise comparisons within and between *cpn*60 subgroups. A) Plot based on 2,278 comparisons with 68 aligned *cpn*60 universal target sequences (552 bp). The long tail of the intra-subgroup distribution is explained by low identity of outliers NR010 and JCP8481A with the rest of subgroup A. B) Plot based on ANIm (average nucleotide identity using MUMmer) including 780 comparisons with 40 whole genome sequences. Tail is due to low identity of JCP8481A/B with the rest of subgroup A. C) Plot based on inter-strain correlation coefficient of tetranucleotide signature frequencies (780 comparisons, 40 genomes), with correlation values >0.99 between strains from the same *cpn*60 subgroup. Small peak at ~0.98 indicates comparisons between JCP8481A/B and other subgroup A genomes. D) Plot based on 16S rRNA gene sequences (231 comparisons with 22 sequences of 1408bp), showing overlap of inter- and intra-subgroup percent identity comparisons for this gene.

Comparisons of pairwise similarity distributions of *cpn*60 UT sequences for all available *G*. *vaginalis* isolates results in a bimodal distribution of inter- and intra-subgroup similarity (Fig 2A), confirming that isolates in each subgroup are phylogenetically distinct from isolates in other subgroups. Interestingly, the lowest values in the intra-subgroup distribution correspond to comparisons between the most phylogenetically distant branch of subgroup A (NR010 and JCP8481A/B) and other subgroup A isolates (Fig 2A). Pairwise Average Nucleotide Identity using MUMmer (Fig 2B) and tetranucleotide values (Fig 2C) calculated for 40 whole genome sequences provide additional support for separate species within *G*. *vaginalis*, that cannot be reliably distinguished using 1408 bp of the 16S rRNA gene (Fig 2D).

### Evaluation of *cpn*60 UT typing in relation to other published typing schemes

**Fig 3:**
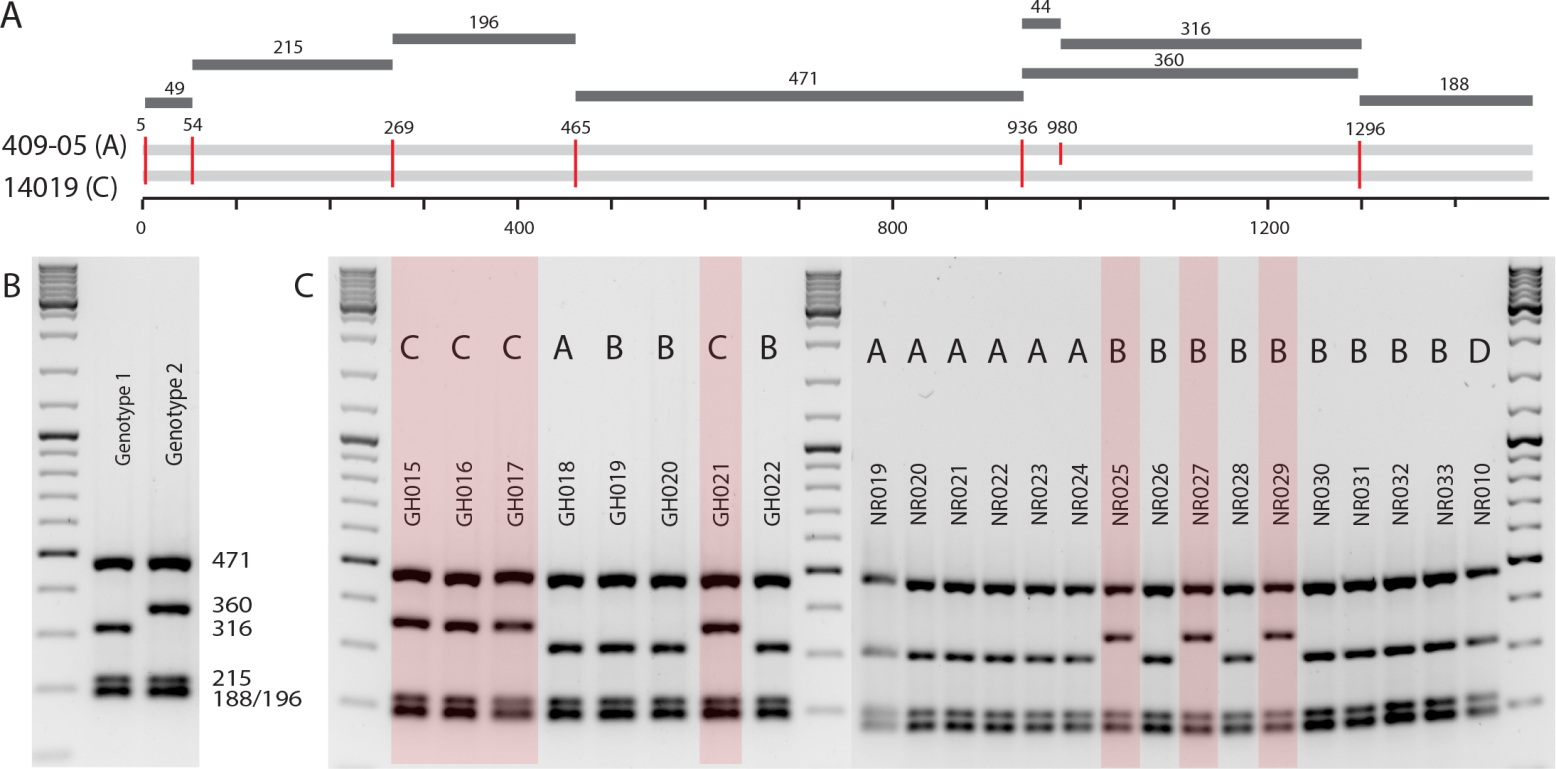
Amplified ribosomal DNA restriction genotyping analysis of *G*. *vaginalis* isolates. A) Model of *Taq*I (T^CAG) restriction sites in 16S rRNA PCR products of subgroup A (409–05) and subgroup C (ATCC14019), B) Sizes of fragments associated with each ARDRA genotype, C) ARDRA profiles for selected *G*. *vaginalis* isolates, showing consistent profiles for subgroups A and C and inconsistent profiles for subgroup B. Lanes containing genotype 1 profiles are un-shaded; lanes containing genotype 2 profiles are highlighted in red.

In order to evaluate performance of *G*. *vaginalis* subgroup typing using the *cpn*60 UT, we also performed previously published molecular typing assays on all 112 isolates in this study. First, we conducted genotyping of all isolates by amplified ribosomal DNA restriction analysis (ARDRA) based on amplification of 16S rRNA gene sequences and digestion of the resulting products with *Taq*I (Fig 3). All isolates fell into two ARDRA genotypes, with all *cpn*60 subgroup A isolates (36/36) belonging to ARDRA genotype 1 (characterized by band sizes of 188, 196, 215, 316 and 471 bp), while all subgroup C isolates (35/35) belonged to ARDRA genotype 2 (characterized by band sized of 188, 196, 215, 360 and 471 bp) (Table 1, Fig 3). Subgroup B and D isolates had both ARDRA genotypes, with 24/33 and 5/8 isolates with genotype 1 and 9/33 and 3/8 isolates with genotype 2 in subgroups B and D, respectively. Therefore, each ARDRA genotype observed in this study corresponded to three *cpn*60 subgroups (ARDRA genotype 1 with subgroups A/B/D, and ARDRA genotype 2 with subgroups B/C/D). A third, previously reported ARDRA genotype [4] was not detected in any isolate.

**Table 1:** Summary of molecular and phenotypic characteristics for 112 isolates of *G*. *vaginalis*.

	*cpn*60 subgroup			
	A (36)	B (33)	C (35)	D (8)
**Country of origin**				
Canada (40)	12 (30%)	11 (28%)	16 (40%)	1 (3%)
Belgium (18)	6 (33%)	6 (33%)	6 (33%)	—
Kenya (54)	18 (33%)	16 (27%)	13 (24%)	7 (13%)
**ARDRA**^1^ **Pattern**				
Genotype 1	36 (100%)	24 (73%)	—	5 (63%)
Genotype 2	—	9 (17%)	35 (100%)	3 (37%)
**Subgroup real-time PCR**^2^ **(hydrolysis probes)**				
Subgroup A	35 (97%)	—	—	—
Subgroup B	—	23 (70%)	—	—
Subgroup C	—	—	35 (100%)	—
Subgroup D	—	—	—	6 (75%)
No assay positive	1 (3%)	10 (30%)	—	2 (25%)
**Subgroup real-time PCR**^2^ **(SYBR green)**				
Subgroup A	30 (83%)	—	—	—
Subgroup B	—	20 (61%)	—	—
Subgroup C	—	—	35 (100%)	—
Subgroup D	—	—	—	8 (100%)
No assay positive	6 (17%)	13 (39%)	—	—
**Sialidase gene presence**^3^^,^*				
Conventional PCR (Sia-F/R)	1 (3%)	33 (100%)	35 (100%)	8 (100%)
SYBR green (GVSI-F/R)	1 (3%)	33 (100%)	35 (100%)	8 (100%)
**Sialidase activity**^4^^,^*				
Filter spot assay	—	33 (100%)	3 (9%)	—
Quantitative fluorometry	—	33 (100%)	3 (9%)	—

^1^Amplified ribosomal DNA restriction analysis

^2^Subgroup-targeted real-time PCR

^3^PCR assays targeting the putative sialidase A

^4^Fluorescence assays using 2'-(4-methylumbelliferyl)-α-D-N-acetylneuraminic acid to detect sialidase activity.

*Differences across *G*. *vaginalis* subgroups are statistically significant by Fisher’s exact test (p<0.0001).

We also compared *cpn*60 UT subgroups to recently published clade-targeted real-time PCR assays intended to differentiate *G*. *vaginalis* clades 1–4 in vaginal samples [9]. These assays were based on subgroup-specific sequences derived from whole genome sequence analysis. Our analysis shows that these assays were specific in all cases, since no assay designed to detect one subgroup detected an isolate belonging to a different subgroup (Table 1, S1 Fig). All isolates defined as *cpn*60 subgroup C were detected using the assays designed to detect them. However, sensitivity was limited since several subgroup B and D isolates, as well as a single subgroup A isolate (NR010), were negative in all assays (Table 1, S1 Fig). These findings were confirmed by repeating real-time PCR assays using duplicate cultures, indicating that subgroup-specific sequences identified for real-time PCR assay development were not found in all isolates. Further work will be required to improve sensitivity of these assays.

### Sialidase gene presence and activity in *G*. *vaginalis* subgroups

**Fig 4:**
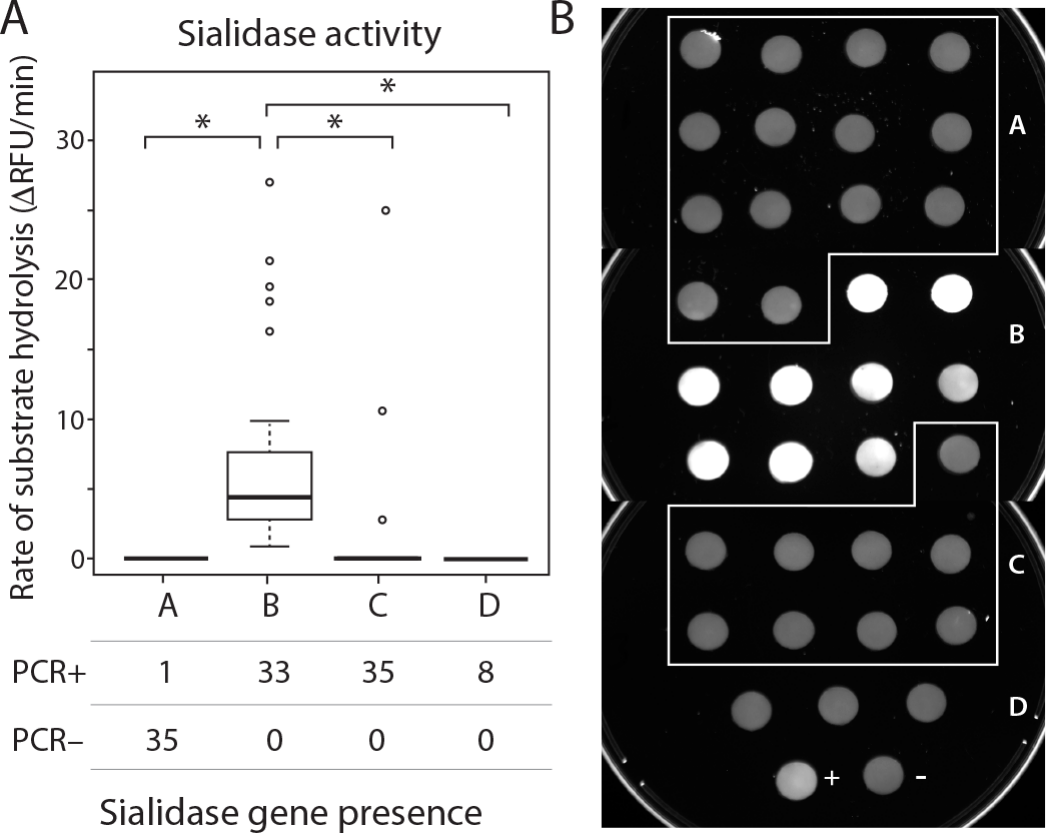
Detection of sialidase gene and sialidase activity in *Gardnerella* isolates. A) Quantitative fluorometry of adjusted sialidase activity (rate of increase in fluorescence adjusted for optical density of culture) confirms that all subgroup B isolates and 3/36 subgroup C isolates are sialidase positive, and all subgroup A and D isolates are sialidase negative. This difference is statistically significant by non-parametric Kruskal-Wallis test (p<0.001), and significant pairwise differences by Mann Whitney U test (p<0.001) are indicated with an asterisk. Phenotypic results are not concordant with PCR detection of the putative sialidase gene, which is also significantly different across subgroups by Fisher’s exact test (p<0.0001). B) Sialidase activity as measured by the filter spot assay, showing that only subgroup B isolates are sialidase positive. Sialidase positive control is strain W11 and negative control is ATCC 14018.

In order to detect the presence of the gene for sialidase activity in our isolates, we applied two previously published assays for detecting the putative sialidase A gene of ATCC 14019 (Genbank Protein Accession YP_003985295) [5, 6]. All subgroup B, C and D isolates were sialidase gene positive using both assays, and all but one subgroup A isolate (NR010) was sialidase gene negative (Table 1, Fig 4A). These findings were confirmed by repeating assays using duplicate cultures. Presence/absence of the putative sialidase gene across groups was significantly different by Fisher’s exact test (p<0.0001).

Surprisingly, gene presence was not predictive of actual sialidase activity using both a qualitative (positive/negative) filter spot assay and highly sensitive quantitative assay based on a fluorescent substrate (Fig 4 and S2 Fig). All subgroup B isolates were sialidase gene positive and activity positive (Table 1, Fig 4). In contrast, all subgroup C isolates were sialidase gene positive but only three isolates, all from Belgium, were sialidase activity positive (Table 1, Fig 4). This finding was confirmed using duplicate cultures. All subgroup A and D isolates were sialidase activity negative, despite detection of the sialidase gene in one subgroup A isolate (NR010) and in all subgroup D isolates. The difference in sialidase activity (positive/negative) across *G*. *vaginalis* subgroups was statistically significant by Fisher’s exact test (p<0.0001, Table 1). When quantitative values for sialidase activity among subgroups were compared, a significant difference was detected by non-parametric Kruskal-Wallis test (p<0.001). Subsequent pairwise comparisons showed that subgroup B had significantly higher activity than other subgroups (Mann Whitney U test, p<0.001)(Fig 4). Complete results for all 112 *Gardnerella* isolates described in this study are provided in S2 Table.

## Discussion

*Gardnerella vaginalis* was first described in 1953 [21], and soon after described as the causative agent of a new kind of vaginitis (*Haemophilus vaginalis* vaginitis) [22], now known as bacterial vaginosis (BV). Despite decades of research, the specific etiology of BV and the role of *Gardnerella* in its clinical and subclinical manifestations remain controversial and poorly defined [2, 23]. Despite well-known phenotypic diversity [3], the possibility that *G*. *vaginalis* may actually consist of several species with distinct roles in BV pathogenesis has only recently been investigated [5, 7, 8]. Studies based on 16S rRNA variable-region targeted sequencing have known biases against detecting *Gardnerella* at all [24, 25], much less resolving subgroups, although one early deep sequencing study did distinguish four *G*. *vaginalis* subgroups based on a single nucleotide difference in variable region 6 of the 16S rRNA gene [26]. Previous studies based on *Taq*I restriction digest of 16S rRNA PCR products indicated the presence of at least three genotypes [5, 7]. In this study, ARDRA pattern was consistent within all isolates in subgroup A (ARDRA genotype 2) and C (ARDRA genotype 1), however subgroup B and D isolates had either ARDRA genotype. ARDRA genotype 3 was not observed in this study or in another recent study of a smaller number of isolates [6]. In contrast, *cpn*60 sequence clearly distinguishes four *G*. *vaginalis* subgroups [25, 27–30], corresponding exactly to whole genome-based analyses. This correspondence between *cpn*60 sequence and whole genome analyses has previously been shown for clostridia and *Enterococcus* [31, 32].

Bimodal distribution of pairwise ANIm values based on 40 whole genome sequences is consistent with the suggested bacterial species level cut-off of 95–96% [19], corroborated by bootstrap values [33] and high correlation of tetranucleotide signature frequencies within *cpn*60 subgroups [19]. Although molecular criteria for designation of *G*. *vaginalis* subgroups as separate species have arguably been met, identification of additional phenotypic properties that consistently resolve these subgroups is required before a formal proposal of reclassification can be made.

The strong correspondence between *cpn*60 subgroups and quantitative PCR results using recently described assays based on subgroup-specific genes in whole genome sequences [9], demonstrates the utility of both approaches. However, one subgroup A isolate and several subgroup B and D isolates were negative by all primer sets. Since these assays were designed to detect and quantify the four subgroups in vaginal samples simultaneously, our observations indicate that some organisms within subgroups A, B and D would not be captured. For example, NR010 shares a valid node with other subgroup A isolates, but differed from all other subgroup A isolates since it was not detected by any subgroup-specific real-time PCR assay, and was positive for the putative sialidase gene. It is only 88% similar to other subgroup A isolates (the trailing left-hand tail on the intra-subgroup density curve), and also 88% identical to the other outliers, JCP8481A and JCP8481B [14]. If confirmed in other isolates, this observation indicates that NR010 and JCP8481A/B may represent two additional molecular subgroups of *Gardnerella* in addition to the four that have already been described.

We have previously shown that subgroup B and C isolates, but no subgroup A and only some subgroup D isolates, were positive for the putative sialidase gene [8]. In this study, we show that this gene is detectable by PCR in all subgroup B, C and D isolates, and in one subgroup A isolate. Our initial investigations revealed that only subgroup B isolates from Kenyan and Canadian samples were sialidase activity positive using the qualitative filter spot test. In a recent study where sialidase activity was determined quantitatively [14], all subgroup B isolates and a single subgroup C isolate were sialidase positive. In this study, all subgroup B isolates and three subgroup C isolates were sialidase positive. The greater sensitivity of the quantitative assay allows us to conclude that most subgroup C isolates and all subgroup A and D isolates are sialidase activity negative and not weakly positive, despite presence of the putative sialidase gene.

Sialidase activity in *G*. *vaginalis* was first described in 1984 [34], however an explicit link between the translated product of the putative gene sequence targeted by PCR assays used in this study and actual enzymatic activity has yet to be confirmed for this organism. Although one previous study did report that all sialidase gene positive strains were also sialidase activity positive [5], this finding was not corroborated by the present study. A lack of sialidase activity in isolates containing the putative gene could be explained by the presence of an alternative gene encoding this activity or other factors abrogating gene expression. Differential sialidase production by *G*. *vaginalis* subgroups has direct clinical significance since detection of sialidase activity in vaginal fluid is currently used as a commercial diagnostic for BV [35]. Sialidase and other mucolytic enzymatic activities in vaginal fluid are likely detrimental to the protective mucous layer and have been proposed to play a role in recurrent BV and pre-term birth [1]. Determination of whether *G*. *vaginalis* subgroups are also ecologically distinct (i.e. occupy different niches in the female reproductive tract), and thus differentially associated with clinical status will require further epidemiological investigations where *G*. *vaginalis* subgroup prevalence and abundance can be observed in the context of the entire microbiome [30]. Phenotypic properties such as sialidase activity could result in *G*. *vaginalis* having a direct effect on the overall composition of the community, leading to characteristic associations of *G*. *vaginalis* subgroups with other particular vaginal bacterial species or consortia. For example, structured polymicrobial biofilms containing primarily *Gardnerella* have been extensively observed in women with persistent BV [12], and sialidase activity has been associated with biofilm formation [5, 36].

In conclusion, our findings have confirmed previous suggestions that new species designations are warranted for better resolution of *G*. *vaginalis* [7, 8]. The *cpn*60 universal target sequence offers a powerful alternative to existing methods for differentiating *G*. *vaginalis* subgroups in cultured isolates and deep sequencing libraries [30]. Ongoing and future studies are aimed at elucidating the clinical significance of sialidase activity in *G*. *vaginalis* subgroups.

## Supporting Information

S1 Fig Quantitative PCR using two sets of assays to detect *cpn*60 subgroup designations A-D. Results for hydrolysis probe assays are shown on left and results for SYBR green assays are shown on right. Final relative fluorescence units (RFU) were calculated as the mean RFU of the final ten PCR cycles minus the standard deviation of the mean. Values greater than 800 were considered positive.
(PDF)

S2 Fig Sialidase activity detection. Detection of sialidase activity by cleavage of fluorescent substrate bound to sialic acid, using a filter spot assay (left) and quantitative fluorometry (right). Note wide variation in rate of substrate hydrolysis and comparability between quantitative and qualitative measures of identical isolates.
(PDF)

S1 Table Primers and probes used in this study. (PDF)

S2 Table Phylogenetic and phenotypic characteristics of *G*. *vaginalis* isolates. (PDF)
