# Supplementary figures and images for: Gardnerella vaginalis Subgroups Defined by cpn60 Sequencing and Sialidase Activity in Isolates from Canada, Belgium and Kenya
Source: PLoS One. 2016 Jan 11;11(1):e0146510. doi: 10.1371/journal.pone.0146510 (PMC4709144; doi:10.1371/journal.pone.0146510)

Hydrolysis probes

SYBR green

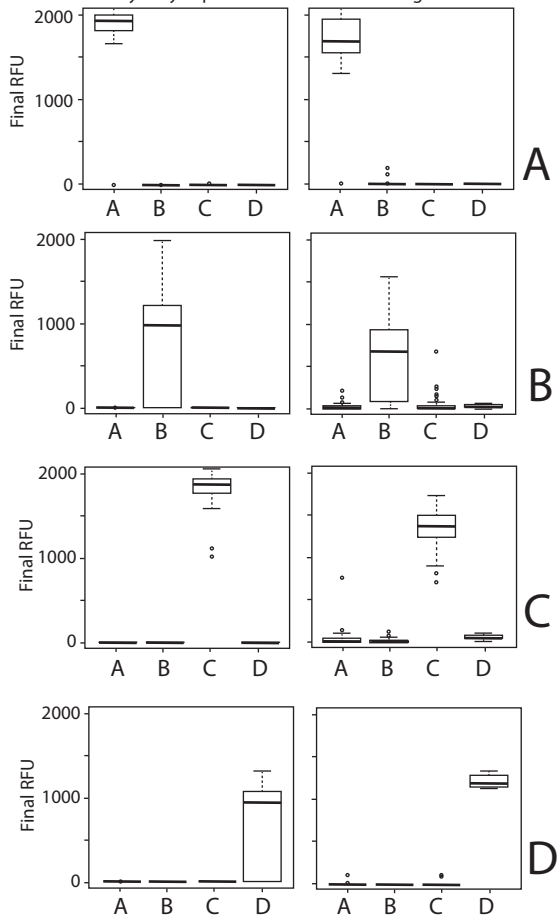

Supplement: S1 Fig — Results for hydrolysis probe assays are shown on left and results for SYBR green assays are shown on right. Final relative fluorescence units (RFU) were calculated as the mean RFU of the final ten PCR cycles minus the standard deviation of the mean. Values greater than 800 were considered positive. (PDF) [file pone.0146510.s001.pdf]

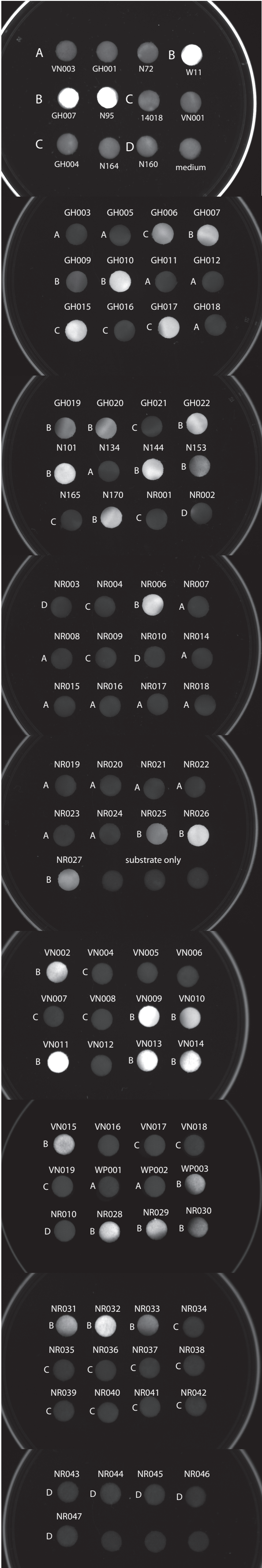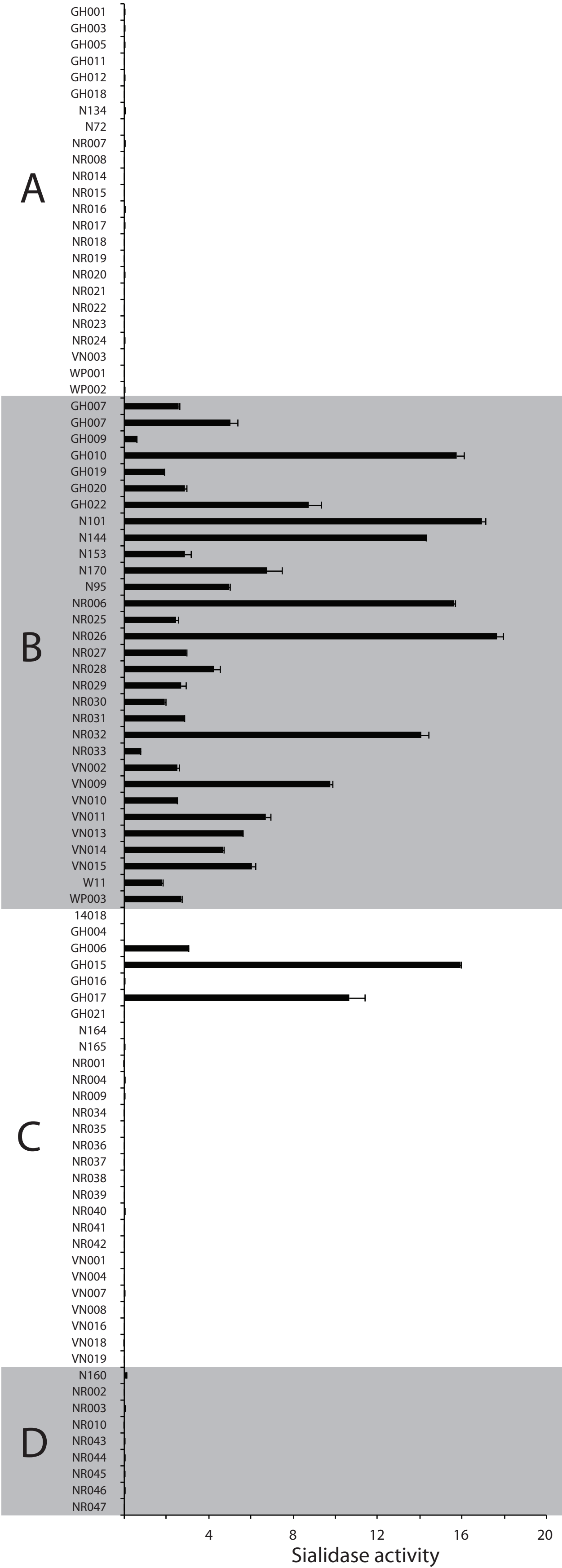

Supplement: S2 Fig — Detection of sialidase activity by cleavage of fluorescent substrate bound to sialic acid, using a filter spot assay (left) and quantitative fluorometry (right). Note wide variation in rate of substrate hydrolysis and comparability between quantitative and qualitative measures of identical isolates. (PDF) [file pone.0146510.s002.pdf]
