# Supplementary material for: Gardnerella vaginalis Subgroups Defined by cpn60 Sequencing and Sialidase Activity in Isolates from Canada, Belgium and Kenya
Source: PLoS One. 2016 Jan 11;11(1):e0146510. doi: 10.1371/journal.pone.0146510 (PMC4709144; doi:10.1371/journal.pone.0146510)
Supplement: S1 Table — (PDF) [file pone.0146510.s003.pdf]

S1 Table. Primers and probes used in this study

| Purpose | Assay | Target strain | Length | Target | Protein ID | Primer/Probe | Sequence | Ref. |
|---|---|---|---|---|---|---|---|---|
| Subgroup A detection | Hydrolysis Probes | 409-05 | 74 | chloride transporter | WP_012914241 | Gv4_cic_S | CCTACGCAAGCTCCAGACGAC | [6] |
| | | | | | | Gv4_cic_AS | ACAAGTTGCACTCTTCGAGCTGG | |
| | | | | | | Gv4_cic_TM | HEX-ACTCGGCTGAAGCACACCACCACT-BHQ1 | |
| | SYBR Green | 409-05 | 139 | allantoate amidohydrolase | WP_012914179 | Gv4_all_S | CACGCTGGCACAACAATGATG | |
| | | | | | | Gv4_all_AS | TTGGAACTACGCTGATTCTACCG | |
| Subgroup B detection | Hydrolysis Probes | 00703Bmash | 124 | hypothetical protein | EIK82797 | Gv2_hyp_S | GCAAAGCAGACTGAGCGTATTAG | |
| | | | | | | Gv2_hyp_AS | GTAATAATCAGGCTCCTCATCGC | |
| | | | | | | Gv2_hyp_TM | HEX-CGCAGGCGCTCGCATAACAGTGCA-BHQ1 | |
| | SYBR Green | 00703Bmash | 137 | Cellulosome anchoring protein | EIK82557 | Gv2_cel_S | GCTTGGGGTTCATATGGTGATGG | |
| | | | | | | Gv2_cel_AS | TCTTTATCAGACACGCCCTTAGC | |
| Subgroup C detection | Hydrolysis Probes | ATCC14019 | 139 | putative alpha-fucosidase | YP_003985210 | Gv1_fuc1_S | CCAGTCATAAGTTTGCGTTTTACC | |
| | | | | | | Gv1_fuc1_AS | TGGCACTGGCAAAGTTTACAAC | |
| | | | | | | Gv1_fuc1_TM | FAM-CTCGCCGCAAGCACCATCAAGCCA-BHQ1 | |
| | SYBR Green | ATCC14019 | 108 | galactokinase | YP_003986287 | Gv1_galK_S | TTCTAGATTATTCGCCGCCAAATC | |
| | | | | | | Gv1_galK_AS | TTGCGATGTGTTGAAGGTAATGC | |
| Subgroup D detection | Hydrolysis Probes | 00703Dmash | 142 | Thioredoxin | EIK84834 | Gv3_thi_S | TTCTGCTTCTTCTGCTATTTGCTG | |
| | | | | | | Gv3_thi_AS | TTCGTTGACTTTTGGGCAACATG | |
| | | | | | | Gv3_thi_TM | FAM-CGGTCCGTGCCGTTCATTTGGTCC-BHQ1 | |
| | SYBR Green | 00703Dmash | 149 | alpha-beta hydrolase fold protein | EIK85650 | Gv3_a-b_S | TGATTACGCTCACGCTCTCG | |
| | | | | | | Gv3_a-b_AS | CGGCAACAGCTTTAGGAAGAAG | |
| Sialidase gene detection | PCR | ATCC14019 | 703 | sialidase A | YP_003985295 | Sia1-F | ATGGAACGTCGTTCAACGAAG | [9] |
| | | | | | | Sia1-R | GATACGCGTTTTATGTCTCTTGC | |
| | SYBR Green | ATCC14019 | 163 | sialidase A | YP_003985295 | GVSI-F | GACGACGGCGAATGGCACGA | [8] |
| | | | | | | GVSI-R | AGTCGCACTCCGCGCAAGTC | |
| 16S rDNA-based typing | PCR / restriction digest (ARDRA) | ATCC14019 | 1483 | 16S rRNA | Gene ID: 9903746 | GV10F | GGTTCGATTCTGGCTCAG | [8] |
| | | | | | | GV10R | TACCTTGTTACGACTTCGTCCCA | |
