# Supplementary material for: Gardnerella vaginalis Subgroups Defined by cpn60 Sequencing and Sialidase Activity in Isolates from Canada, Belgium and Kenya
Source: PLoS One. 2016 Jan 11;11(1):e0146510. doi: 10.1371/journal.pone.0146510 (PMC4709144; doi:10.1371/journal.pone.0146510)
Supplement: S2 Table — (PDF) [file pone.0146510.s004.pdf]

S2 Table. Phylogenetic and phenotypic characteristics of *Gardnerella vaginalis* isolates

Strain	*cpn*60 subgroup	ARDRA pattern	Real-time PCR for subgroup detection[1]								Sialidase gene presence and activity			
			Hydrolysis probe assays				SYBR green assays							
			A	B	C	D	A	B	C	D	PCR[2]	SYBR[1]	Spot[3]	Fluorometry[4]
GH001	A	1	1829	0	0	6	1945	0	63	0	0	0	0	0
GH003	A	1	2088	0	0	0	2088	20	81	0	0	17	0	0
GH005	A	1	1954	0	0	0	1954	211	107	0	0	0	0	0
GH011	A	1	1936	0	0	0	1936	27	85	0	0	8	0	0
GH012	A	1	2218	0	0	0	2218	0	133	0	0	0	0	0
GH018	A	1	1764	0	0	0	1311	0	10	0	0	6	0	0
N115	A	1	1487	0	0	0	0	0	0	0	0	0	0	0
N134	A	1	1768	0	0	0	1398	61	19	0	0	0	0	0
N72	A	1	1660	0	0	0	1929	0	47	0	0	0	0	0
NR007	A	1	1903	0	0	0	1903	0	26	0	0	0	0	0
NR008	A	1	1970	0	0	0	1970	0	761	0	0	96	0	0
NR010	A	1	0	0	0	0	0	0	23	0	1	1055	0	0
NR014	A	1	1893	0	0	0	1893	71	7	22	0	0	0	0
NR015	A	1	1811	0	0	0	0	128	9	0	0	74	0	0
NR016	A	1	2037	0	0	0	1540	0	0	0	0	0	0	0
NR017	A	1	1979	0	0	0	1979	0	0	0	0	0	0	0
NR019	A	1	1948	0	0	0	1948	0	46	0	0	0	0	0
NR020	A	1	1949	0	0	0	1949	20	35	0	0	15	0	0
NR021	A	1	2052	0	0	0	2052	12	11	0	0	0	0	0
NR022	A	1	1823	0	0	0	1823	36	26	0	0	0	0	0
NR023	A	1	1903	0	0	0	1570	36	0	0	0	0	0	0
NR024	A	1	1977	0	0	0	1608	2	0	0	0	0	0	0
NR045	A	1	1708	7	0	0	0	40	82	98	0	50	0	0
NR046	A	1	1773	0	0	0	0	0	12	0	0	0	0	0
VN003	A	1	1779	0	0	0	2086	27	78	106	0	0	0	0
WP001	A	1	1818	0	0	0	1572	0	0	0	0	0	0	0
WP002	A	1	1923	0	0	0	1671	0	0	0	0	0	0	0
WP006	A	1	1818	0	0	1	1485	0	0	0	0	0	0	0
WP011	A	1	1910	0	0	0	1478	0	0	0	0	0	0	0
WP015	A	1	2010	0	0	0	1570	0	0	0	0	0	0	0

S2 Table. Phylogenetic and phenotypic characteristics of *Gardnerella vaginalis* isolates

Strain	*cpn*60 subgroup	ARDRA pattern	Real-time PCR for subgroup detection[1]								Sialidase gene presence and activity			
			Hydrolysis probe assays				SYBR green assays							
			A	B	C	D	A	B	C	D	PCR[2]	SYBR[1]	Spot[3]	Fluorometry[4]
WP016	A	1	2080	0	0	0	1689	0	0	0	0	0	0	0
WP020	A	1	2081	0	0	0	1719	0	0	0	0	0	0	0
WP021	A	1	2015	0	0	0	1723	0	3	0	0	0	0	0
WP022	A	1	1929	0	0	0	1587	0	0	0	0	0	0	0
WP024	A	1	2094	0	0	0	1649	0	0	0	0	0	0	0
WP026	A	1	2085	0	0	0	1687	0	0	0	0	0	0	0
GH007	B	1	0	1233	0	0	10	789	82	0	1	1163	1	5.61
GH009	B	1	0	1191	0	0	3	735	84	0	1	1935	1	0.91
GH010	B	1	0	1151	0	0	0	935	40	0	1	2138	1	26.96
GH019	B	1	0	980	0	0	1	795	38	0	1	1800	1	2.15
GH020	B	1	0	1027	0	0	0	857	4	0	1	2063	1	3.67
GH022	B	1	0	920	0	0	0	0	26	0	1	1755	1	9.78
N101	B	2	0	0	0	0	0	517	0	0	1	1734	1	18.39
N117	B	2	0	1984	0	0	0	1564	0	0	1	1190	1	2.85
N144	B	2	0	975	0	0	0	1104	0	0	1	1731	1	21.23
N153	B	2	0	0	0	0	0	135	0	0	1	1408	1	2.38
N170	B	1	0	1900	0	0	0	0	0	0	1	1627	1	7.30
N95	B	1	0	0	0	0	0	1155	32	0	1	1123	1	8.00
NR006	B	1	0	0	0	0	184	677	62	0	1	1828	1	16.29
NR025	B	2	0	0	0	0	0	14	0	0	1	1123	1	3.57
NR026	B	1	0	0	0	0	0	701	0	0	1	1868	1	21.27
NR027	B	2	0	0	0	0	0	90	0	0	1	1143	1	2.78
NR028	B	1	1	1524	0	0	0	1027	0	0	1	1784	1	5.02
NR029	B	2	0	0	0	0	0	82	121	0	1	1213	1	2.30
NR030	B	1	0	1368	0	0	0	1024	0	0	1	1994	1	1.97
NR031	B	1	0	0	0	0	0	999	1	0	1	2013	1	2.92
NR032	B	1	0	795	0	0	0	1056	0	0	1	1795	1	19.45
NR033	B	1	0	0	0	0	0	93	0	0	1	1866	1	1.11
VN002	B	1	0	1446	0	0	0	0	28	0	1	2075	1	2.80
VN009	B	1	0	1011	0	0	0	883	0	0	1	1710	1	7.68

S2 Table. Phylogenetic and phenotypic characteristics of *Gardnerella vaginalis* isolates

Strain	*cpn*60 subgroup	ARDRA pattern	Real-time PCR for subgroup detection[1]								Sialidase gene presence and activity			
			Hydrolysis probe assays				SYBR green assays							
			A	B	C	D	A	B	C	D	PCR[2]	SYBR[1]	Spot[3]	Fluorometry[4]
VN010	B	1	0	1095	0	0	0	830	14	0	1	1615	1	2.80
VN011	B	1	0	1356	0	0	0	75	0	0	1	1856	1	6.85
VN013	B	1	0	949	0	0	0	168	0	0	1	1831	1	4.98
VN014	B	1	0	1185	0	0	0	260	0	0	1	1817	1	4.38
VN015	B	1	0	935	0	0	0	184	19	0	1	1734	1	5.58
W11	B	1	0	1442	0	0	0	1032	10	0	1	1025	1	4.57
WP003	B	2	0	1151	0	0	0	1527	0	0	1	1142	1	2.57
WP027	B	2	0	1045	0	0	103	0	0	0	1	1127	1	3.14
WP028	B	1	0	1475	0	0	0	748	0	0	1	1442	1	2.23
GH004	C	2	0	0	1661	0	0	17	813	0	1	1142	0	0.00
GH006	C	2	0	0	1878	0	0	0	1422	0	1	1361	1	2.97
GH015	C	2	0	0	2047	0	0	0	1332	86	1	1441	1	26.60
GH016	C	2	0	0	2034	0	0	0	1380	0	1	1220	0	0.00
GH017	C	2	0	0	1784	0	0	0	1467	0	1	1670	1	10.83
GH021	C	2	0	0	1934	0	0	0	1136	0	1	1471	0	0.00
N165	C	2	0	0	1012	0	0	0	1119	0	1	1157	0	0.00
NR001	C	2	0	0	1877	0	0	0	1339	0	1	1709	0	0.00
NR004	C	2	1	0	1999	0	0	0	1485	0	1	1906	0	0.00
NR009	C	2	0	0	1989	0	0	0	1346	0	1	1781	0	0.00
NR034	C	2	0	0	1875	0	0	0	1194	0	1	1966	0	0.00
NR035	C	2	0	0	1864	0	0	253	1499	0	1	1875	0	0.00
NR036	C	2	1	0	1899	0	0	165	1387	107	1	1975	0	0.00
NR037	C	2	0	0	1783	0	0	0	1594	0	1	2190	0	0.00
NR038	C	2	0	0	1679	0	0	76	1481	0	1	2050	0	0.00
NR039	C	2	0	0	1781	0	0	99	1547	0	1	2041	0	0.00
NR040	C	2	1	0	1757	0	0	32	1542	0	1	1751	0	0.00
NR041	C	2	0	0	1874	0	0	232	1670	0	1	2048	0	0.00
NR042	C	2	0	0	1934	0	0	53	1532	0	1	1981	0	0.00
VN001	C	2	0	0	1579	0	0	143	1282	0	1	1196	0	0.00
VN004	C	2	0	0	1635	0	0	0	1534	0	1	1862	0	0.00

S2 Table. Phylogenetic and phenotypic characteristics of *Gardnerella vaginalis* isolates

Strain	*cpn*60 subgroup	ARDRA pattern	Real-time PCR for subgroup detection[1]								Sialidase gene presence and activity			
			Hydrolysis probe assays				SYBR green assays							
			A	B	C	D	A	B	C	D	PCR[2]	SYBR[1]	Spot[3]	Fluorometry[4]
VN007	C	2	0	0	1798	0	0	41	1738	0	1	1422	0	0.00
VN008	C	2	0	0	1761	0	0	145	1669	0	1	1607	0	0.00
VN016	C	2	0	0	1878	0	0	0	952	0	1	1115	0	0.00
VN018	C	2	0	0	1831	0	0	0	1102	0	1	1450	0	0.00
VN019	C	2	0	0	1772	0	0	0	1302	0	1	1192	0	0.00
WP007	C	2	1	0	1770	0	0	0	1234	0	1	1307	0	0.00
WP008	C	2	0	0	1978	0	0	0	1407	0	1	1451	0	0.00
WP009	C	2	1	0	1945	0	0	0	1252	0	1	1454	0	0.00
WP010	C	2	0	0	1922	0	0	0	1287	0	1	1467	0	0.00
WP014	C	2	0	0	1765	0	0	0	1293	0	1	1463	0	0.00
WP018	C	2	1	0	1958	0	0	0	1430	0	1	1496	0	0.00
WP019	C	2	1	0	2060	0	0	0	1239	0	1	1358	0	0.00
WP023	C	2	1	0	2041	0	0	0	1310	0	1	1399	0	0.00
WP025	C	2	29	0	1122	0	0	0	1314	0	1	1455	0	0.00
N160	D	1	0	0	0	1115	0	0	0	1272	1	569	0	0.00
N161	D	1	0	0	0	1166	0	0	0	1290	1	659	0	0.00
NR002	D	2	0	0	0	0	0	0	58	1171	1	1442	0	0.00
NR003	D	1	0	0	0	1316	0	35	55	1354	1	702	0	0.00
NR043	D	1	0	0	0	1074	0	402	109	1217	1	992	0	0.00
NR044	D	1	0	0	0	961	0	0	0	1652	1	458	0	0.00
NR047	D	2	0	0	0	0	0	16	9	1140	1	1535	0	0.00
WP012	D	2	0	0	0	1083	0	0	0	1680	1	853	0	0.00

[1] Values are mean relative fluorescent units minus standard deviation of last 10 PCR cycles, all negative values adjusted to 0.

[2] Presence of band in Sia-F/R PCR assay, 0=Negative, 1=Positive

[3] Visible fluorescence of culture in presence of substrate on filter paper spots under UV light, 0=Negative, 1=Positive

[4] Values are change (slope) of fluorescence over time of cultures in presence of substrate measured on a FLx800 fluorometer, adjusted to 0.1 OD_{595}
